# SERUM PROGRANULIN AS A POTENTIAL BIOMARKER FOR FRAILTY IN CHINESE OLDER ADULTS

**DOI:** 10.1093/geroni/igac059.2998

**Published:** 2022-12-20

**Authors:** Pan Liu, Yun Li, Shijie Li, Yaxin Zhang, Yu Song, Tong Ji, Ying Li, Lina Ma

**Affiliations:** Xuanwu Hospital Capital Medical University, Beijing, Beijing, China (People’s Republic); Xuanwu Hospital Capital Medical University, Beijing, Beijing, China (People’s Republic); Xuanwu Hospital Capital Medical University, Beijing, Beijing, China (People’s Republic); Xuanwu Hospital Capital Medical University, Beijing, Beijing, China (People’s Republic); Xuanwu Hospital Capital Medical University, Beijing, Beijing, China (People’s Republic); Capital Medical University Xuanwu Hospital, Beijing, Beijing, China (People’s Republic); Xuanwu Hospital Capital Medical University, Beijing, Beijing, China (People’s Republic); Xuanwu Hospital Capital Medical University, Beijing, Beijing, China (People’s Republic)

## Abstract

**Background:**

Frailty increases the risk of adverse health outcomes in older adults. Progranulin is a secreted glycoprotein involved in regulating various biological processes. A few studies have investigated the relationship between progranulin and frailty in middle-aged and older populations; however, different perspectives exist. We aimed to evaluate the association of progranulin with frailty in older Chinese adults.

**Methods:**

We included 265 older in-patients who were divided into the frail (n=107) and non-frail (n=158) groups according to the FRAIL scale. Serum IL-6, CXCL-10, and progranulin levels were assayed. Spearman’s correlation analysis and logistic regression models were used to analyze the association of serum biomarkers with frailty, and the ROC was used to evaluate the diagnostic progranulin value for frailty.

**Results:**

The frail group was older, had lower BMI, higher prevalence of coronary heart disease, worse grip strength and walking speed, and higher serum levels of IL-6, CXCL-10, and progranulin, than the non-frail group. Progranulin levels were negatively correlated with grip strength and positively correlated with IL-6 and CXCL-10. The logistic regression analysis showed that IL-6, CXCL-10, and progranulin were associated with frailty, respectively. Furthermore, progranulin remained associated with frailty after adjusting for age, sex, BMI, smoking, and chronic diseases (OR=1.003, 95%CI=1.001–1.005, p=0.013). The AUC of serum progranulin levels for diagnostic frailty was 0.872 (95%CI=0.829–0.914, p < 0.001).

**Conclusion:**

High serum progranulin levels were observed in frail older in-patients and associated with worse physical function and chronic inflammation. This association indicates that progranulin may be a potential biomarker for frailty.

